# Sex Differences in Emotion Recognition and Working Memory Tasks

**DOI:** 10.3389/fpsyg.2018.01072

**Published:** 2018-06-29

**Authors:** Rahmi Saylik, Evren Raman, Andre J. Szameitat

**Affiliations:** ^1^Department of Psychology, Faculty of Art and Science, Mus Alparslan University, Mus, Turkey; ^2^Department of Life Sciences, Centre for Cognitive Neuroscience, Brunel University London, Brunel University London, Uxbridge, United Kingdom

**Keywords:** gender differences, emotion recognition, visuospatial working memory, executive functions, Cambridge Neuropsychological test battery (CANTAB)

## Abstract

It is proposed that emotional and cognitive functions may be differentiated based on sex. However, it is still unknown whether this assumption could be generalized for all emotional faces and working memory (WM) functions. To examine this, 50 females, and 60 males performed an emotion recognition task, consisting of a series of emotional faces as well as three working memory tasks from Cambridge Neuropsychological test battery (CANTAB); namely, spatial working memory (SWM), stocking of Cambridge (SOC), and intra/extradimensional shifts tasks (IED). The results found that females had faster response times in recognition of both positive and negative faces as compared to males. Furthermore, it was observed that while females were better on SWM task processing, males performed better on IED and four move SOC tasks, illustrating that processing of WM components may differentiate by sex. It has been concluded that emotional and cognitive functions are indeed sensitive to sex differences.

## Introduction

Sex differences regarding cognitive and emotional processing have been of great interest for years. It has been reported that females are more sensitive to emotional and stressful stimuli based on electro-dermal activities and self-report measures during processing of cognitive and emotion tasks as compared to males (Kring and Gordon, 1998; Kret and De Gelder, 2012). Previous empirical research suggests affective and cognitive processing may be sex-differentiated such as in emotion recognition and working memory (WM) processing due to these factors, i.e., higher emotionality and stress level (Kring and Gordon, 1998; Montagne et al., 2005). A highly complex process such as facial expression recognition likely requires an organizing mechanism such as WM in order to maintain and coordinate the different perceptual and memory-related neural components involved in the recognition of facial expressions (Streit et al., 1999). In addition, several studies postulate that responses to facial emotional stimuli are mediated by attentional processes (McKenna et al., 2001; Holmes et al., 2003; Pourtois et al., 2006) and that the interaction between attention and WM is bidirectional (e.g., Gonzalez-Garrido et al., 2007).

Experimental studies of sex differences in facial emotion recognition and WM tasks have often reported contradictory findings. Several studies suggest that females outperform males in specific WM tasks such as spatial working memory (SWM) (Owen et al., 1990; Duff and Hampson, 2001; Kaufman, 2007; Harness et al., 2008), and emotion recognition (McClure, 2000; Hall and Matsumoto, 2004; Montagne et al., 2005; Kret and De Gelder, 2012; Sawada et al., 2014). However, other studies report either no difference or indicate an advantage for males in emotion processing and WM tasks (Grimshaw et al., 2004; Derntl et al., 2010; Sawada et al., 2014; Voyer et al., 2017). It is therefore imperative to shed further light on the apparent disparity within the literature by taking into consideration both emotion recognition and WM performance whilst exploring sex differences. The current study aims to investigate sex differences at a broader level by employing both an emotion recognition task and several WM measures that are each associated with a different function of WM in a relatively large sample.

One approach to investigating emotional processing in sex differences utilizes a facial emotion recognition test. The test involves the presentation of various emotional stimuli involved in perception, attention, and memory (Edwards et al., 2002; Chan et al., 2009). Previous emotion recognition studies have used drawings, cartoons, video clips, and photographs as stimuli (Edwards et al., 2002). However, the validity of specific emotional stimuli (e.g., drawings, cartoons) has been disputed (Edwards et al., 2002). In this regard, the emotional picture set from the affective series of Ekman and Friesen (1976) is considered to be an essential resource in the investigation of emotion recognition and is widely used throughout the domain due to the robust validity of this set of pictures (Edwards et al., 2002).

Previous empirical research has often reported that higher level emotionality and stress levels lead to attentional biases toward emotional stimuli due to an increased level of alertness during the presentation of emotional stimuli (Haas et al., 2006; Doty et al., 2013; Andric et al., 2016). In this context, females are reported to exhibit higher levels of emotional and stress responses (Kring and Gordon, 1998; Kret and De Gelder, 2012), it would be reasonable to predict such attentional biases toward emotional stimuli in females compared to males. Despite few contradictions, meta-analytic reviews suggest that the majority of studies related to emotion recognition demonstrate that females are faster in recognition of emotional faces (McClure, 2000; Kret and De Gelder, 2012). For example, Hampson et al. (2006) performed an emotion recognition task by using real facial emotional pictures and found that females were faster in recognizing both positive and negative emotional faces. Furthermore, Vassallo et al. (2009), reported that females were faster than males in recognition of happy faces. Conversely, Sawada et al. (2014) investigated sex differences in rapid detection of emotional icons, i.e., drawing lines of faces. The authors found no difference between females and males both in response times and accuracy. In line with this, Hoffmann et al. (2010) also reported no differences between females and males in recognition of real emotional faces with higher density. A potential explanation for such inconsistency in emotion recognition tasks may concern discrepancies like stimuli and task domains (Kret and De Gelder, 2012). Specifically, several studies that investigate sex differences in emotion recognition task used emotional icons and video clips or pictures with either higher or lower emotional density and reported contradictory findings (McClure, 2000; Hoffmann et al., 2010; Sawada et al., 2014).

Working memory, i.e., control of attention, storing and manipulation of information, is an essential cognitive domain to explore sex differences. According to Baddeley's (2000) model, WM can be divided into several components (i.e., Visuospatial sketchpad (VSSP), storing visual, and spatial information, and Phonological loop (PL), storing auditory information) and a central executive function (CES, supervises memory store, control, and manipulate information). It has been suggested that CES has divisible functions such as inhibition (suppressing task irrelevant stimuli), switching (shifting attention from one task to another), planning (evaluation and selection of task relevant information), and updating (maintenance of task related information) (Baddeley, 1996; Miyake et al., 2000). Regarding sex differences in WM task performance, sex differences in SWM are well investigated (Owen et al., 1990; Duff and Hampson, 2001; Kaufman, 2007; Voyer et al., 2017). For example, in a recent study, SWM tasks were performed by females and males samples, and they found that females outperformed males as evidenced by lower error rates in females (Duff and Hampson, 2001; Harness et al., 2008). On the other hand, several studies report a male advantage on SWM tasks (Bosco et al., 2004; Kaufman, 2007; Voyer et al., 2017). For example, Kaufman (2007), investigated sex differences by employing several SWM tasks (i.e., simple span tasks that is similar to Corsi block, and rotation tasks, and verification block tasks which are rather complex tasks). The author concluded that males were significantly better on complex SWM tasks but not on simple span tasks which are associated with the visuospatial component of WM (Kaufman, 2007). In addition, few studies suggested no sex differences on Corsi block tasks (Farrell Pagulayan et al., 2006). One potential reason for such contradiction may be the use of different task domains as previous research indicates that performing either computerized or physical cognitive tasks leads to contradictory results in studies of sex differences (Cooper, 2006; Kay, 2006).

Despite accumulated empirical studies related to sex differences in SWM tasks, the knowledge about sex differences related to other components of WM such as executive functions is sparse. Two studies that have explored the executive functioning domain both support sex differences: stop-signal tasks (Thakkar et al., 2014) and the n-back task (Schoofs et al., 2013). Both studies found that males performed better on specific outcome measures of the tasks. However, these studies only provide limited information as they investigate executive function by focusing on a singular task. Working memory studies suggested that CES has distinct functions such as switching (shifting attention from one task to another), inhibition (i.e., suppression of task irrelevant stimuli), and planning (sequencing order of stimuli) (Baddeley, 1996; Miyake et al., 2000). It has been reported that CES functions such as inhibition, switching, and planning can be assessed as the significant functions of CES (Ozonoff and Jensen, 1999; Miyake et al., 2000; Ozonoff et al., 2004). These functions require higher cognitive effort as compared to other sub-functions such as monitoring or storage systems during task processing (Eysenck and Derakshan, 2011). Therefore, to understand sex differences at a broader level concerning WM processing, it has to be tested in specific tasks that are each associated with a different function.

It is therefore vital to further investigate the role of sex differences in both emotion and WM tasks concurrently. Furthermore, as previous empirical studies often report that vulnerability to stress and emotionality is higher in females than in males (McClure, 2000; Montagne et al., 2005; Fugate et al., 2009), it is also important to consider the effect of emotionality and stress on cognitive abilities and emotional processes (Eysenck, 1963; Eysenck and Calvo, 1992; Eysenck and Derakshan, 2011). For example, several studies found that emotionality and stress cause worry related thoughts which interfere with processing of WM and cause attentional biases toward emotional stimuli (MacLeod and Rutherford, 1992; Osorio et al., 2003). Therefore, investigating sex differences in emotion recognition and WM tasks will facilitate a greater understanding of sex-related performance on these domains. Also, it would be useful to clarify whether any potential effect due to sex differences in one domain (e.g., emotion recognition task) will be revealed in another domain (e.g., WM tasks).

Theoretical models related to individual differences suggest that emotionality, stress, and arousal levels are strongly integrated, and this often negatively influences functions of WM (Eysenck, 1963; Eysenck and Calvo, 1992; Eysenck and Derakshan, 2011). It has been reported that stress-related activities impair WM components differently (Eysenck and Calvo, 1992; Eysenck and Derakshan, 2011). In more detail, the findings demonstrated that CES functions such as switching and inhibition would have significant impairments due to stress-related activities as compared to VSSP (Eysenck, 1963; Eysenck and Calvo, 1992; Eysenck and Derakshan, 2011). In this context, if females are more sensitive to emotional and stressful stimuli than males, there should also be reported impairment in CES functions. To further examine this, we test for sex-related differences using standardized WM tasks found in the Cambridge Neuropsychological Tasks Automated Battery (CANTAB; Sahakian and Owen, 1992). For example, IED set shifting tasks is a computerized version of Wisconsin Card Sorting test and it is strongly associated with switching and inhibition functions (Ozonoff et al., 2004). Stocking of Cambridge (SOC) is analogous to the Tower of London test and is strongly associated with planning function (Ozonoff et al., 2004). Finally, the SWM task is analogous to the Corsi Block test and is used to measure the function of VSSP (Owen et al., 1990; Ozonoff et al., 2004). Employing these subtasks will allow investigating sex differences in WM task processing in detail because of the increasing task difficulty of these subtests.

The present study aims to investigate sex differences in facial emotion recognition and WM tasks at a broader level in a relatively large sample from a novel perspective. Specifically, facial pictures with varied emotional intensities from the affective series of Ekman and Friesen (1976) which is well validated across the world were selected for the emotion recognition tasks (Ekman et al., 1971; Ekman and Friesen, 1976). The reason for that is to increase the validity of data regarding emotion processing in sex differences by employing universally recognized emotions (Ekman et al., 1971). Regarding WM tasks, Cambridge Neuropsychological Tasks Automated Battery (CANTAB) was used as the test tool, and from this battery, IED (highly similar to the WCST, assessing switching and inhibition) was chosen for testing sex differences on switching and inhibition functions. Furthermore, to examine sex differences on another CES function, i.e., planning, the SOC test (highly similar to TOH, assessing planning) was chosen. Moreover, SWM (similar to Corsi Block and assessing VSSP) was selected to replicate previous studies which show sex differences. Memory loads in the SWM and SOC tasks were manipulated from very easy to very difficult to examine any potential effect of sex differences in a broader perspective. To our knowledge, this is the first study which investigates sex differences at a broader level regarding the use of both distinct WM domains and facial emotion recognition tasks.

## Methods

### Participants

One hundred ten participants (50 female, 60 males, i.e., sex were matched as 45% females and 55% males) aged 18–40 (males: *M* = 22.97 years, *SD* = 4.17; females: 21.50, *SD* = 5.32) from Brunel University London took part the study. Based on self-report measures, the participants have no past or current psychiatric or neurologic disorders. Based on the reports of Edinburgh Handedness Inventory (Oldfield, 1971), all participants were right-handed (Oldfield, 1971). In all participants, predicted IQ was higher than 70 according to National Adult Reading Test II (NART II) (NART II; Nelson, 1982). All participants were either native English speakers or fluent in English. Participants gave a written informed consent just before participation in experiments. The experiments took 1-h, and the participants received £10 for their participation. The Department of Life Sciences ethics committee at Brunel University approved the study.

### Emotional face recognition task

In the current study, to investigate sex differences in emotional processing, emotional face pictures were selected from the affect series of Ekman and Friesen (1976). This series of emotional faces consists of six facial emotions (sad, disgusted, surprised, fearful, angry, and happy) with the intensity of emotions varying from the full emotion (100%) to 25%. In total, there were 144 emotional facial pictures which are equally divided for each emotion, i.e., each facial emotion had 24 pictures. For the experimental presentation, E-Prime (18-2.0.8.22), Psychology Software Tools, Philadelphia, USA) was used to control the appearance of pictures. In the task, emotional faces were displayed for 500 ms on the computer screen. After this time interval, the names of six emotions were presented. Participants were required to respond to the appropriate emotion that described the preceding face by using the mouse provided to click on the emotion. Participants had to make their response in the given time, otherwise missing responses or responses by accident (too fast < 250 ms; too long >6,000 msec) were taken as incorrect responses. Outcome measures were mean response times and the total number of correct trials for each category of emotion.

### Working memory tasks

Three WM tasks which are Stoking of Cambridge (SOC), Spatial Working Memory task (SWM), Intra-Extra Dimensional Shift task (IED) were selected from CANTAB (http://www.cambridgecognition.com/cantab/) to investigate sex differences in various functions of WM processes.

#### Stockings of cambridge (SOC)

In the SOC task, there are two configurations which each consisted of 3 colored balls, one on the top and the other on the bottom of the touchscreen. Participants are required to look at the two configurations, and they must regulate the balls in the bottom configuration to make it same as the top configuration. To do this, they need to move the balls in a specific order because they must match the goals with a certain number of moves (between two and five moves). Also, participants were told that they should think about the planning of appropriate moves and then they should move a ball. Task difficulty is varied from two to five moves. While in the most straightforward condition they must complete the task with two moves, in the most challenging condition they need to complete the task within five moves. Outcome measures were mean response times and the number of correct trials for each condition. In other words, the average response times and the correct number of trials were taken separately for 2, 3, 4, and 5 moves condition (Ozonoff et al., 2004; Torgersen et al., 2012).

#### Spatial/visual working memory task (SWM)

The SWM task is associated with VSSP storage capacity of WM. In this task, participants were presented with a number of blue boxes in a spatial array with an empty column on the right side of the array. The boxes contain yellow hidden tokens and participants were required to find the tokens by touching the boxes. Hence, participants were required to search for hidden tokens (e.g., touching blue token which is moved to empty column). When they find a token in a box, it will not contain another token again so that they should search in other boxes where a token hasn't been found yet. Also, when the participants touch a box which hasn't contained a token yet, they should search the token again until they find it. For efficient task performance, participants should remember the location of the boxes where a token has been found and the other boxes where the token hasn't been found. Thus, they will complete the task successfully with fewer error rates. Task difficulty is increased from searching 4 to 6 and 8 boxes. Outcome measures in this task are “between search errors” meaning the number of times a participant revisits a box in which a token has already been found within a given trial. “Within search errors” referring to the number of times a participant revisits a box already found to be empty during the same search as well as “total errors” refers the average number of errors that occurred in 4, 6, and 8 boxes (Owen et al., 1990).

#### Intra-extra dimensional shift task (IED)

IED is similar to the Wisconsin card sorting test (Grant and Berg, 1948), and it is associated with the switching and inhibition functions of the central executive system (Ozonoff et al., 2004). In this task, participants were presented with a set of compounded stimuli which consists of two colored shapes and two ramified lines. In this task, participants always see four objects on the screen, a compounded set of a colored shape and a white line on the left, and a compounded set of a colored shape and a white line on the right. There are two primary rules for task performance. In the first rule, participants should ignore the lines and respond based on the shapes and in the second rule they should ignore shapes and respond based on the lines. Task performance is hence based on rule learning so that participants should touch a compounded stimulus to determine whether the touched stimuli is correct. Participants received feedback that indicated whether the response was correct or not to learn the correct rule based on the feedback received. The rule is altered after six correct responses without notification, and participants have to discover the new rule based on the feedback. The outcome measures are total errors (i.e., mean errors throughout the task), total trials (i.e., the number of trials that are performed), successfully completed stages (i.e., the number of successfully completed conditions) and EDS errors (errors that occurred during extradimensional shifts).

### Procedure

Before the experiment, all participants read and signed the informed consent form. Subsequently, participants completed the following questionnaires: Edinburgh handedness inventory, a self-assessment questionnaire of past or current history of psychiatric or neurological illness, a verbal IQ test (NART). Based on the results of the questionnaires, the exclusion criteria were as follows: IQ < 70, color blindness, use of psychoactive medication, and a history of past or current neurological or psychological disorders. By employing such exclusion criteria, potential confounding effects due to these factors should be reduced as emotional and cognitive functioning may be influenced by those factors. The experiments consisted of two phases; WM and emotion recognition phases. Finally, participants completed the experiments individually in a quiet cubicle room. Presentation of the two phases (emotion recognition task and WM tasks) was randomized so that participants either performed emotion recognition task at first or WM tasks. For the emotion recognition task, a computer which runs E-prime was used. For the WM tasks, CANTAB was used, and the three WM subtasks were again randomized for each participant. All tasks were first practized to eliminate sensorimotor or comprehension difficulties. The participants were instructed verbally from a script. On average, the study took 1 h.

## Results

Multivariate analyses of covariance MANCOVA was calculated when examining sex differences in the emotion recognition and WM tasks. The significance of all effects was reported at *p* < 0.05 unless otherwise stated. The grouping variable was sex, i.e., females and males. The controlling variable was age and predicted intelligence (NART). The testing variables were the task conditions.

### Emotion recognition task

The results regarding sex differences in emotion recognition tasks showed that females were faster on responding to faces accurately whereas males and females had comparable scores of accurately recognized emotions (see Table 1). In more detail, females were significantly faster than males on recognition of sad faces after controlling for participant's age [RT, *F*_(1, 109)_ = 9.59; *p* = 0.003], ${\overset{´}{\eta }}^{2}$ = 0.084; disgusted faces [RT, *F*_(1, 109)_ = 5.39; *p* = 0.019], ${\overset{´}{\eta }}^{2}$ = 0.051; surprised face [RT, *F*_(1, 109)_ = 5.33; *p* = 0.023], ${\overset{´}{\eta }}^{2}$ = 0.048; fearful faces [RT, *F*_(1, 109)_ = 4.50; *p* = 0.036], ${\overset{´}{\eta }}^{2}$ = 0.041; angry faces [RT, *F*_(1, 107)_ = 4.11; *p* = 0.048], ${\overset{´}{\eta }}^{2}$ = 0.037; happy faces [RT, *F*_(1, 109)_ = 3.70; *p* = 0.054], ${\overset{´}{\eta }}^{2}$ = 0.034. However, regarding the number of correctly recognized faces, males and females had similar performance as evident by non-significant results through all pairwise comparisons after controlling age and predicted intelligence [all accuracy *F*_(1, 109)_ < (largest: 3.01/lowest:.16), all *p* > (largest: 0.69/lowest: 0.072)]. The results indicate that while females are faster than males on recognition of emotional faces, males and females had comparable performance on the number of correctly recognized faces.

**Table 1:** Shows mean and standard deviations (SD) for correct hits and response times in emotion recognition tasks in males and females.

**Outcome measures**	**Sex**	**N**	**Mean**	**Std. Deviation**
Angry accuracy	Male	60	0.49	0.14
	Female	50	0.50	0.13
Disgusted accuracy	Male	60	0.37	0.17
	Female	50	0.39	0.12
Fearful accuracy	Male	60	0.45	0.16
	Female	50	0.46	0.13
Happy accuracy	Male	60	0.81	0.10
	Female	50	0.82	0.08
Sad accuracy	Male	60	0.65	0.14
	Female	50	0.69	0.14
Surprised accuracy	Male	60	0.59	0.13
	Female	50	0.64	0.11
Angry response time	Male	60	1172.51	486.17
(correct trials)	Female	50	1021.58	300.71
Disgusted response	Male	59	1429.57	745.05
time (correct trials)	Female	50	1151.32	428.90
Fearful response time	Male	60	1488.50	617.30
(correct trials)	Female	50	1266.68	450.85
Happy response time	Male	60	814.92	256.23
(correct trials)	Female	50	730.87	194.77
Happy response time	Male	60	1121.26	389.57
(correct trials)	Female	50	907.20	323.35
Surprised response	Male	60	1289.41	578.53
time (correct trials)	Female	50	1059.76	436.58

In addition, when examining sex differences in emotion recognition regarding overall response times (including correct and error trials all together) after controlling age and predicted intelligence, the results were still similar as follows; sad face, [RT, *F*_(1, 109)_ = 11.28; *p* = 0.001], ${\overset{´}{\eta }}^{2}$ = 0.097; disgusted faces [RT, *F*_(1, 109)_ = 8.24; *p* = 0.005], ${\overset{´}{\eta }}^{2}$ = 0.073; surprised face [RT, *F*_(1, 109)_ = 5.14; *p* = 0.025], ${\overset{´}{\eta }}^{2}$ = 0.047; fearful faces [RT, *F*_(1, 109)_ = 6.28; *p* = 0.014], ${\overset{´}{\eta }}^{2}$ = 0.056; angry faces [RT, *F*_(1, 107)_ = 6.82; *p* = 0.010], ${\overset{´}{\eta }}^{2}$ = 0.061; happy faces [RT, *F*_(1, 109)_ = 3.40; *p* = 0.061], ${\overset{´}{\eta }}^{2}$ = 0.032. Despite of numerical changes in the results, it seems males and females still had comparable performance on the overall response times of recognized faces (see Table 2).

**Table 2:** Shows mean and standard deviations (SD) for overall response times in emotion recognition tasks in males and females.

**Outcome measures**	**Gender**	**N**	**Mean**	**Std. Deviation**
Angry response time (overall)	Male	60	1492.4768	563.86775
	Female	49	1230.0592	359.86629
Disgusted response time (overall)	Male	60	1349.6865	520.97205
	Female	49	1089.0137	326.35737
Fearful response time (overall)	Male	60	1551.6577	527.17507
	Female	49	1289.9147	464.34989
Happy response time (overall)	Male	60	1016.6553	315.26002
	Female	49	906.1422	258.32186
Happy response time (overall)	Male	60	1380.5305	476.51534
	Female	49	1092.5400	344.42982
Surprised response time (overall)	Male	60	1449.3802	568.80444
	Female	49	1200.7206	439.08138

### Working memory tasks

MANCOVA results showed that sex differences in only certain conditions of WM tasks whereas no sex differences were found in the majority of conditions in WM tasks as evident by all *p*-values >0.05 after controlling age and predicted intelligence (NART).

In line with this, females and males showed similar performance on other conditions of SWM tasks which are associated with spatial WM [all *F*_(110)_ < (largest: 2.90/lowest: 0.02), all *p* > (largest: 0.96/lowest: 0.14)]. The results indicate that females had similar scores regarding average of total errors and performance in searching 4, 6 and 8 boxes (see Table 3).

**Table 3:** Shows mean and SD in SWM task outcome measures for females and males.

**Outcome measures**	**Sex**	**N**	**Mean**	**Std. Deviation**
SWM 4 boxes	Male	60	1.13	2.11
	Female	50	0.34	1.23
SWM 6 boxes	Male	60	4.75	5.25
	Female	50	3.76	5.12
SWM 8 boxes	Male	60	12.83	10.69
	Female	50	13.40	9.12
SWM tot errors	Male	60	18.93	16.22
	Female	50	18.02	13.74

The results regarding SOC task performance shows that females and males had similar performance in all outcome measures of SOC except for the 4 moves condition after controlling age and predicted intelligence (see Table 4). The result showed that males were significantly faster than females in the 4 moves SOC task [*F*_(1, 109)_ 4.62 *p* = 0.034], ${\overset{´}{\eta }}^{2}$ = 0.043. However, females and males had similar performance in all other conditions. This was evident by non-significant results through all pairwise comparisons for mean of RTs in SOC moves (2, 3, and 5) [all *F*_(1, 109)_ < (largest: 1.59/lowest: 0.05), all *p* > (largest: 0.90/lowest: 0.21)]. Likewise, mean number of moves showed non-significant results: [all *F*_(1, 109)_ < (largest: 3.01/lowest: 0.05), all *p* > (largest: 0.77/lowest: 0.09)]. The results indicate that females and males differ in response time on the 4 moves SOC condition whereas they did not differ on SOC task performance regarding in other conditions.

**Table 4:** Shows mean and SD in response times and the correct number of responses in SOC outcome measures for females and males.

**Outcome measures**	**Sex**	**N**	**Mean**	**Std. Deviation**
2 moves	Male	60	1840.55	2451.26
	Female	50	1863.53	907.35
3 moves	Male	60	4222.57	4778.06
	Female	50	5034.78	2869.08
4 moves	Male	60	7541.25	5687.12
	Female	50	10112.18	6920.78
5 moves	Male	60	10368.28	7609.93
	Female	50	13098.48	13553.38
Mean 2 moves	Male	60	2.11	0.45
	Female	50	2.01	0.07
Mean 3 moves	Male	60	3.16	0.41
	Female	50	3.14	0.33
Mean 4 moves	Male	60	5.25	0.83
	Female	50	5.13	0.96
Mean 5 moves	Male	60	6.16	1.39
	Female	50	6.33	1.33
Minimum total moves	Male	60	9.32	1.95
	Female	50	9.24	1.76

Finally, the results regarding IED task showed sex differences in one outcome measure after controlling age and predicted intelligence (EDS errors) [*F*_(1, 109)_, 3.80, *p* = 0.05]. Females made more errors during processing of EDS shifts which is strongly associated with switching and inhibition functions (see Table 5). However, females and males did not differ significantly in all other outcome measures [all *F*_(1, 109)_ < (largest: 1.88/ lowest: 0.74), all *p* > (largest: 0.39/lowest: 0.17)]. The results indicate that females and males had completed a similar number of trials, completed stages and average of total errors.

**Table 5:** Shows mean and SD in IED task outcome measures for females and males.

**Outcome measures**	**Sex**	**N**	**Mean**	**Std. Deviation**
EDS errors	Male	60	7.08	8.498
	Female	50	11.50	10.037
IED stages completed	Male	60	8.72	0.691
	Female	50	8.56	0.812
IED total errors	Male	60	15.30	9.677
	Female	50	18.10	11.814
IED total trials	Male	60	77.42	16.702
	Female	50	80.42	20.127

## Discussion

In the present study, sex differences in the processing of facial emotion recognition and WM tasks were investigated. The results showed significant differences between females and males in the processing of facial emotion recognition tasks in all emotional faces whereas sex differences were found only in some of the WM conditions. Furthermore, females were faster than males in recognition of all emotional faces as evident by shorter responses in recognition of sad, disgusted, surprised, fearful, angry and happy faces. Additionally, in WM tasks while males and females had similar performance in searching boxes in the SWM task, males performed better in the EDS outcome measure of IED tasks and four moves SOC tasks.

The first aim of the study was to investigate sex differences in processing of facial emotion recognition tasks. Present results are consistent with the empirical studies which showed females are faster than males on recognition of emotional faces for both positive and negative faces (McClure, 2000; Vassallo et al., 2009; Hoffmann et al., 2010; Kret and De Gelder, 2012). It has been indicated that females are more emotional and stressful which lead attentional biases toward emotional stimuli (Kring and Gordon, 1998; Fugate et al., 2009; Schoofs et al., 2013). Indeed, there is empirical evidence that shows that factors such as emotionality and stress prioritize encoding of emotional cues to detect emotional stimuli rapidly. It is suggested that these factors influence the focus of attention by putting the cognitive system in alert mode toward emotional stimuli (Osorio et al., 2003; Haas et al., 2006; Eysenck and Derakshan, 2011; Andric et al., 2016).

Present results are consistent with previous findings related to emotion recognition tasks (Kring and Gordon, 1998; Montagne et al., 2005; Hampson et al., 2006; Hoffmann et al., 2010; Kret and De Gelder, 2012). For instance, Hampson et al. (2006) examined whether sex differences exist in the processing of facial emotion recognition task and found that females were indeed faster than males in recognition of both positive and negative faces. This is in line with the conclusion that female performance was superior in emotion recognition. Also, a few studies have reported no sex difference in emotion recognition (Sawada et al., 2014). It has been suggested that the reason for such contradictions might be nature of emotional stimuli because the studies which found no sex differences often used drawing lines such as icons or stimuli with high emotional intensities (McClure, 2000; Hoffmann et al., 2010; Sawada et al., 2014). However, in the current study, real emotional faces were selected from affect series of Ekman and Friesen (1976) who tested and validated these faces regarding emotion intensity and recognition across the world. Thus, the results seem to be more objective and valid as compared to the studies which failed to show sex differences by illustrating female superiority in the facial emotion recognition task.

Theoretically, it has been suggested that such differences between males and females are due to different socialization processes (Brody, 1985) and hormonal secretions which may lead different brain activations in males and females (Speck et al., 2000; Kret and De Gelder, 2012). For instance, Kret and De Gelder (2012) reviewed sex differences in processing of emotional stimuli. They suggested that females are more emotional than males as evident by higher skin conductance results when viewing emotional stimulus. Males and females are differing regarding brain structures due to different hormonal secretion since childhood (Speck et al., 2000). In line with that, electrophysiological studies showed distinct activations of the visual cortex in P1 and N1, while men were associated with right hemisphere activations women were associated with bilateral activity in women (Proverbio et al., 2006). It has been found that left hemisphere activation for female and right hemisphere activations in males were found during the presentation of emotional expressions (Lee et al., 2002). These differences between males and females may be lead regarding sex differences in emotion recognition as in the current findings.

The second aim of the study was to investigate sex differences in WM memory processing. The results showed sex differences in particular conditions of each WM task whereas no sex differences were found in WM tasks when averaging their performance across each task. It seems that while males and females did not significantly differ in conditions of SWM which is associated with VSSP component of WM, males seem to be better in some conditions of executive functions which is associated with planning (SOC task) and switching and inhibition functions (IED task). However, this difference between the performance of females and males is small because when assessing the task performance overall, they did not differ. This is in line with metanalytic reviews which show females and males vary on specific WM tasks with a small but significant difference (Kaufman, 2007; Voyer et al., 2017).

Importantly, to resolve the contradiction found between previous findings sex differences in individual WM tasks such as SWM, the current study manipulated task demand from easy and challenging conditions in SWM and SOC tasks as some individuals may show different performance on only specific difficulties (Eysenck, 1963). In this context, males showed better performance on the four move SOC condition whereas no significant sex differences were found on conditions of the SWM task. Thus, these results suggest sex differences could be observed in particular conditions of certain WM tasks which are associated with executive functions The results regarding the SWM task are consistent with previous empirical research which showed no sex differences on SWM tasks (Farrell Pagulayan et al., 2006). Current results seem to be consistent with the study of (Kaufman, 2007) as well because of in that study while males and females significantly differ on complex SWM tasks, no sex differences were found on simple span tasks which are analogous to SWM tasks in the current study. It should be noted that in the study of Kaufman (2007) complex SWM tasks consisted of two tasks (e.g., Verbal tasks and rotation task) and participants had to perform two tasks in rapid succession as a dual task. It is known that performing two tasks in a rapid succession often associate with executive functions as well (Baddeley, 1996; Jiang, 2004). However simple span task is mainly taken as a measure of visuospatial sketchpad that is a component of WM (Owen et al., 1990; Kaufman, 2007). In this perspective, the current study is in line with (Kaufman, 2007) because while no differences were found in SWM tasks, there were small but significant sex differences in executive function tasks (IED and SOC tasks) in favor of men.

On the other hand, the studies which suggested females superiority in SWM tasks (Duff and Hampson, 2001; Harness et al., 2008) have not controlled for age and intelligence in the statistical analyses. These empirical findings indicate that females had fewer errors in SWM tasks as compared to males. As it has been indicated WM components could be divisible in prefrontal regions so that these components may differ based on sex as well (Duff and Hampson, 2001; Harness et al., 2008). For instance, females have been shown to be better on perceptual speed and accuracy on visual tasks (Born et al., 1987; Duff and Hampson, 2001). This means that females may faster perceive visual details and encode locations because SWM is associated with VSSP which is storage component of WM (Duff and Hampson, 2001). Therefore, females may better perceive and encode location of information (Duff and Hampson, 2001). In this context, if we do not control for age and predicted IQ, our results in line with this interpretation because females seem to be better on specific conditions of SWM; SWM 4 boxes [*t*_(107)_ = 2.07; *p* = 0.041]. However, despite statistical differences in favor of women in the overall conditions of SWM after controlling age and predicted IQ, the significance disappeared. Therefore, such results could be due to using different statistical approaches.

Furthermore, the results regarding SOC and IED tasks demonstrate male's superiority on certain conditions as compared to females. A similar pattern of these results has been found in the tasks which are associated with executive functions, i.e., stop signal task and n-back tasks (Schoofs et al., 2013; Thakkar et al., 2014). This differentiation in executive functions might be due to higher emotional and stress level in females as compared to males. The theories related to individual differences such as attentional control theory (Eysenck et al., 2007) and dual mechanisms of control (Braver et al., 2007; Braver, 2012) suggest some evidence that emotionality and stress level mainly impairs CES functions while WM storage mostly remains unaffected (Eysenck et al., 2005; Eysenck and Derakshan, 2011). The potential reason for that is CES functions require higher mental effort in the task processing as compared to storage systems (Eysenck and Calvo, 1992; Eysenck et al., 2005; Eysenck and Derakshan, 2011). CES is thought to be involved in attentional control and manipulation of information in addition to supervising WM storages (Baddeley and Hitch, 1974; Baddeley, 1996). It should be noted that while SWM is associated with a storage system of WM, SOC and IED are associated with CES of WM (Ozonoff and Jensen, 1999; Ozonoff et al., 2004). Based on this knowledge, emotionality and stress level in females may lead to differences that favor males during processing of WM tasks which are associated with executive functions.

Sex differences are often reported with statistically significant but small differences (Duff and Hampson, 2001; Harness et al., 2008; Schoofs et al., 2013; Thakkar et al., 2014). The current study is consistent with these studies by showing significant differences in only specific outcome measures whereas overall females and males performed similarly on all WM tasks. Indeed, that would be quite surprising, if the results would show significant differences between females and males in WM performance, instead of current results that show only slight sex differences because both females and males were all healthy individuals and the potential confounding factors such as age and predicted IQ were controlled. There are two reasons in support of this interpretation. First, the current study used a large sample (60 females, 50 males) which could be sufficient to show significant sex differences as compared to past studies. Second, it has been demonstrated that past or current history of psychiatric or neurological disorders such as depression, anxiety or epilepsy, IQ level, use of psychoactive drugs could negatively influence WM processing and thus lead potential confounding effects. Several previous studies did not use such exclusion criteria. Thus, the conclusion indicates there might be the difference between females and males, but this would be somewhat small difference as illustrated by small, significant differences in specific conditions.

To summarize, the current study proposes that while females were faster in recognition of facial pictures and more accurate in the SWM task (assessing VSSP), males had better performance on the four moves SOC task (assessing planning) and a part of the IED task (EDS) (evaluating switching and inhibition). The results regarding emotional recognition task indicate that higher emotionality and stress level in females may lead to attentional biases toward emotional stimuli. Further, the results regarding WM tasks suggest that females were superior in their ability to scan and correctly perceive visual information in the VSSP, whereas males had performed partially better on WM tasks which are associated with CES. The latter finding may be due to higher emotionality and stress disrupting functions of CES in females. Taken together current results demonstrate that emotional and WM processes may differ by sex. While the present research has shown sex differences in emotion recognition and WM processing, future studies need to test sex differences in executive functions further. For instance, sex differences in WM tasks that consist of emotional stimuli should be tested. Also, in the current study, all participants were either native English speakers or fluent in English, however we did not considered their racial profiles. As the cultural differences may influence perception in recognition of emotional stimuli, the continued investigation in terms of sex differences during emotion recognition task should consider racial profiles as well.

In conclusion, the current results indicate that females showed faster response times in recognition of negative and positive faces as compared to males. Further, it has been observed while females were better on SWM task processing, males were better on IED and four move SOC tasks, illustrating that there are likely sex differences in the processing of distinct WM components.

## Author contributions

RS selected the experimental tasks, conducted the experiment, run analyses and write the manuscript. AS supervised in experimental design, checked experimental results and revised the paper and contribute in writing paper. ER revised the paper, contribute discussion, statistical analyses and added more references.

### Conflict of interest statement

The authors declare that the research was conducted in the absence of any commercial or financial relationships that could be construed as a potential conflict of interest.
